# Genomic factors related to tissue tropism in *Chlamydia pneumoniae* infection

**DOI:** 10.1186/s12864-015-1377-8

**Published:** 2015-04-07

**Authors:** Thomas Weinmaier, Jonathan Hoser, Sebastian Eck, Inga Kaufhold, Kensuke Shima, Tim M Strom, Thomas Rattei, Jan Rupp

**Affiliations:** Division of Computational Systems Biology, Department of Microbiology and Ecosystem Science, University of Vienna, 1090 Vienna, Austria; Department of Genome Oriented Bioinformatics, Technical University Munich, 85354 Freising, Germany; Center for Human Genetics and Laboratory Diagnostics Dr. Klein, Dr. Rost and Colleagues, 82152 Martinsried, Germany; Department of Molecular and Clinical Infectious Diseases, University of Luebeck, 23538 Luebeck, Germany; Institute of Human Genetics, Helmholtz Center Munich, 85764 Neuherberg, Germany

**Keywords:** *Chlamydia pneumoniae*, Genome assembly, Comparative genomics, Tissue tropism, SNPs, InDels

## Abstract

**Background:**

*Chlamydia pneumoniae* (*Cpn*) are obligate intracellular bacteria that cause acute infections of the upper and lower respiratory tract and have been implicated in chronic inflammatory diseases. Although of significant clinical relevance, complete genome sequences of only four clinical *Cpn* strains have been obtained. All of them were isolated from the respiratory tract and shared more than 99% sequence identity. Here we investigate genetic differences on the whole-genome level that are related to *Cpn* tissue tropism and pathogenicity.

**Results:**

We have sequenced the genomes of 18 clinical isolates from different anatomical sites (e.g. lung, blood, coronary arteries) of diseased patients, and one animal isolate. In total 1,363 SNP loci and 184 InDels have been identified in the genomes of all clinical *Cpn* isolates. These are distributed throughout the whole chlamydial genome and enriched in highly variable regions. The genomes show clear evidence of recombination in at least one potential region but no phage insertions. The *tyrP* gene was always encoded as single copy in all vascular isolates. Phylogenetic reconstruction revealed distinct evolutionary lineages containing primarily non-respiratory *Cpn* isolates. In one of these, clinical isolates from coronary arteries and blood monocytes were closely grouped together. They could be distinguished from all other isolates by characteristic nsSNPs in genes involved in RB to EB transition, inclusion membrane formation, bacterial stress response and metabolism.

**Conclusions:**

This study substantially expands the genomic data of *Cpn* and elucidates its evolutionary history. The translation of the observed *Cpn* genetic differences into biological functions and the prediction of novel pathogen-oriented diagnostic strategies have to be further explored.

**Electronic supplementary material:**

The online version of this article (doi:10.1186/s12864-015-1377-8) contains supplementary material, which is available to authorized users.

## Background

*Chlamydia* are obligate intracellular bacteria known to infect not only humans, but also reptiles, amoebae, fish and mammals [1]. Beside *C. trachomatis* and *C. psittaci*, *C. pneumoniae* (*Cpn*) is the third chlamydial species with human pathogenic potential, showing the highest infection and seropositivity rates (>70%) amongst them at the age of 65 years [2]. While acute infections mainly affect the upper and lower respiratory tract and clinically impress as pharyngitis or pneumonia, chronic *Cpn* infections have been implicated in the pathogenesis of atherosclerosis and chronic obstructive pulmonary disease (COPD). Both are amongst the most widespread diseases of the elderly and are the first and fourth leading causes of death among the elderly in the industrialized world [3].

Genomic signatures of *Cpn* can be reliably found in atherosclerotic plaques, but also in circulating blood monocytes from patients suffering from acute myocardial infarction [4,5]. Although short-term antimicrobial treatment does not ameliorate cardiovascular disease progression in humans, data from numerous animal studies give supporting evidence that *Cpn* can disseminate from the lung and infect the vasculature using peripheral blood monocytes (PBMC) [6,7]. Only a few studies focusing on genomic differences between respiratory and vascular isolates have been published, none of them was using whole genome analysis of clinical isolates. Thus, it was suggested that respiratory strains contain multiple copies of the *tyrP* gene; whereas, vascular strains contain only a single copy [8]. In contrast, Cochrane et al. later described multiple *Cpn* genotypes in atherosclerotic plaques of carotid endarterectomy specimens by sequencing the VD4 region of the *ompA* gene and a 568 nt fragment spanning the *ygeD* and *urk* genes [9].

It was shown recently by Harris et al., that whole-genome sequencing provides a more accurate scenario of the diversity of clinical *C. trachomatis* isolates than genotyping of single genes that are subject to recombination between different chlamydial biovars [10]. Although most *Cpn* genes are supposed to be less variable, specific regions in the genome baring the potential to vary by homologous recombination and slipped-mispair have been described [11-13]. At that time, however, the available sequence data were not yet sufficient to determine the actual contribution of recombination events to the evolution of *Cpn* strains.

So far, the genomes of only four human pathogenic *Cpn* have been sequenced [14-16]. All of them have been isolated from the respiratory tract. They show a high level of identity in their genome sequence and only few insertions and deletions (InDels) between isolates. We followed the strategy to perform whole-genome sequencing of selected respiratory and non-respiratory *Cpn* isolates to reliably determine the occurrence of single nucleotide polymorphisms (SNPs), InDels and copy number variations (CNVs) in comparison to the already published *Cpn* genomes. We have selected isolates with widespread geographical distribution and sampled over a long period for sequencing, in order to define genomic markers that are most related to the anatomical origin of the respective *Cpn* isolate.

## Results and discussion

### Genome sequencing and assembly

We selected *Cpn* clinical isolates originating from different tissues and spanning a broad range of sampling times and locations as well as an animal isolate as a phylogenetic out-group. From 24 isolates, which were cultivated on HEp-2 cells and sequenced in multiple runs on an Illumina GA II sequencer, 19 yielded a sufficient amount of reads for genome assembly (Table 1). The fraction of chlamydial sequences in the total DNA ranged from 4% (CWL011) to 32% (PB1). Only the GiD isolate DNA could be enriched to 99% chlamydial sequences.

**Table 1:** **Isolate overview**

**Isolate**	**Tissue type**	**Isolation source**	**Continent**	**Country**	**Year of isolation**	**Source**	**Accession**
CWL029	respiratory	throat	Northamerica	USA (Atlanta)	before 1987	NCBI GenBank	NC_000922.1
AR39	respiratory	pharynx	Northamerica	USA (Seattle)	1983	NCBI GenBank	NC_002179.2
J138	respiratory	pharynx	Asia	Japan	1994	NCBI GenBank	NC_002491.1
TW183	conjunctival	conjunctiva	Asia	Taiwan	1965	NCBI GenBank	NC_005043.1
LPCoLN	respiratory	nasal swab (koala)	Australia	Australia	NA	NCBI GenBank	NC_017285.1
CM1	respiratory	sputum	Northamerica	USA (Atlanta)	1991	Black/ATCC	ERS640705
CV14	vascular	coronary artery	Europe	Germany (Mainz)	2002	Maass	ERS640706
CV15	vascular	coronary artery	Europe	Germany (Mainz)	2002	Maass	ERS640707
CWL011	respiratory	throat	Northamerica	USA (Atlanta)	1987/1988	Black/CDC	ERS640708
CWL029c	respiratory	throat	Northamerica	USA (Atlanta)	1991	Black/ATCC	ERS640709
DC9	NA	liver (frog)	Africa	Central African Republic	NA	Sachse	ERS640710
GiD	respiratory	respiratory tract	Europe	Germany (Gießen)	before 1997	Hegemann	ERS640711
H12	respiratory	respiratory tract	Europe	Finland	1991	Saikku	ERS640712
K7	respiratory	respiratory tract	Europe	Finland	1987	Saikku	ERS640713
MUL2216	respiratory	bronchoalveolar lavage	Europe	Germany (Lübeck)	2001	Maass	ERS640714
Panola	respiratory	respiratory tract	Europe	Finland	NA	Saikku	ERS640715
PB1	vascular	PBMC	Europe	Germany (Lübeck)	1997	Maass	ERS640716
PB2	vascular	PBMC	Europe	Germany (Lübeck)	1998	Maass	ERS640717
U1271	respiratory	respiratory tract	Europe	Sweden (Umea)	NA	Boman	ERS640718
UZG1	respiratory	respiratory tract	Europe	Belgium	before 1996	Ossewaarde/CDC	ERS640719
Wien1	vascular	carotid artery	Europe	Austria (Vienna)	1998- 1999	Apfalter	ERS640720
Wien2	vascular	femoral artery	Europe	Austria (Vienna)	1998- 1999	Apfalter	ERS640721
Wien3	vascular	infrarenal artery	Europe	Austria (Vienna)	1998- 1999	Apfalter	ERS640722
YK41	respiratory	nasopharynx	Asia	Japan (Hiroshima)	before 1992	Kanomoto/CDC	ERS640723

Origin of all isolates used in the study. The genomes for the first five isolates are publicly available, genomes of all other isolates were newly sequenced in this study (study accession number: PRJEB8246); sample accessions are given for all newly sequenced genomes; NA: not available.

Mapping the non-human reads from each isolate against the reference isolate CWL029 resulted in 93.7%-99.9% coverage of the reference genome (Table 2). Compared to CWL029, therefore ~1.2 Kb to 77.5 Kb of the newly sequenced genomes consist of polymorphic regions in which no reads could be matched against the reference. In order to further resolve these polymorphic regions, a reference-aided comparative assembly approach was applied.

**Table 2:** **Assembly statistics**

			**Filtering**				**Mapping**				**Comparative assembly**			
**Isolate**	**Read length**	**Total seq. (Mb)**	**Passed filtering (Mb)**	**Passed filtering %**	**Filter criterion**	**Chlamydial reference isolate**	**Mapped total (Mb)**	**Mapped to human (Mb)**	**Mapped to Chlamydia (Mb)**	**Chlamydia reference coverage %**	**Total for comp. assembly (Mb)**	**# Contigs**	**Total assembled nucleotides**	**Average coverage**
CM1	76	2561	1827.9	71.38%	qual, l36	AR39	1662.3	1518.7	143.6	99.9%	309	1	1,229,887	147.0
CV14	74	2094	1747.1	83.44%	qual, l36, mp	CWL029	1703.1	1440.5	262.6	99.9%	307	1	1,228,123	243.0
CV15	36	624	602.0	96.47%	qual, l30	CWL029	498.3	388.9	109.5	99.9%	213	3	1,228,165	86.5
CWL011	76	3061	2040.3	66.65%	qual, l36	CWL029	1954.2	1876.8	77.4	99.9%	163	1	1,228,579	58.3
CWL029c	36	556	545.3	98.07%	qual, l30, mp	CWL029	532.1	463.5	68.6	99.8%	82	7	1,228,575	56.0
DC9	74	1919	1724.6	89.87%	qual, l36, mp	CWL029	1640.8	1511.1	129.7	97.5%	214	85	1,214,883	111.6
GiD	76	1772	1675.8	94.57%	qual, l36, mp	AR39	1610.4	20.8	1589.6	99.9%	1655	2	1,229,850	1,334.6
H12	76	294	259.0	88.11%	qual, l36, mp	CWL029	248.8	233.2	15.7	93.7%	26	147	1,224,589	12.9
K7	74	2344	2137.0	91.17%	qual, l36, mp	CWL029	2076.6	1847.3	229.3	99.9%	290	1	1,228,523	195.2
MUL2216	36	520	510.3	98.14%	qual, l30, mp	CWL029	467.6	420.8	46.8	99.8%	90	7	1,228,567	38.2
Panola	54	1025	912.0	88.98%	qual, l36, mp	CWL029	881.9	837.3	44.6	99.7%	75	13	1,228,514	38.5
PB1	36	464	457.1	98.51%	qual, l30, mp	CWL029	446.4	302.1	144.3	99.9%	155	2	1,228,135	113.2
PB2	74	1806	1612.0	89.26%	qual, l36, mp	CWL029	1564.9	1338.2	226.6	99.9%	274	2	1,228,201	195.5
U1271	74	1623	1494.5	92.08%	qual, l36, mp	CWL029	1462.7	1351.4	111.3	99.9%	143	3	1,228,567	93.9
UZG1	36	472	464.6	98.43%	qual, l30, mp	TW183	389.3	325.1	64.2	99.7%	139	3	1,225,933	52.7
Wien1	76	1241	1150.2	92.68%	qual, l36, mp	CWL029	1120.5	885.5	235.0	99.9%	265	1	1,228,125	195.5
Wien2	74	1955	1784.7	91.29%	qual, l36, mp	CWL029	1722.1	1440.8	281.3	99.8%	344	9	1,228,526	237.8
Wien3	76	3223	1702.8	52.83%	qual, l36	CWL029	1657.0	1344.6	312.4	99.9%	358	1	1,228,576	243.8
YK41	36	549	538.0	97.99%	qual, l30, mp	AR39	522.7	492.1	30.6	99.0%	46	61	1,229,406	25.0

Summary statistics of the processing steps in the assembly of the sequenced isolates. Filter criteria were adjusted to each dataset (qual: quality trimming from 3′ end to a minimal Phred score of 20; l36: minimal read length after trimming of 36 nt; l30: minimal read length after trimming of 30 nt; mp: only paired reads).

The comparative assembly of the non-human reads of the sequenced isolates against the closest available complete reference sequence among the publicly available genomes of *Cpn* produced between 1 and 147 contigs. At least 97.5% of the corresponding reference genome was covered in all of the isolates (Table 2). Compared to the widely used strategy of mapping, comparative assembly also allows reconstructing highly variable regions of the genome, longer insertions and deletions as well as genome rearrangements. In order to examine the correctness of the assembly process, we compared the SNPs derived from our assemblies to results from an earlier study from Rattei et al. [12], in which selected chromosomal regions of the same *Cpn* isolates have already been sequenced. The comparison indicates that 99.5% of the SNPs identified in the previous study were successfully recovered in our current whole-genome assemblies (Additional file 1: Table S1). A test for heterozygous SNPs, which would have indicated different subpopulations, yielded no evidence for the presence of multiple genotypes in any of the isolates (data not shown). Along with these newly sequenced genomes we have also included the publicly available genomes of four clinical *Cpn* isolates (CWL029, AR39, J138 and TW183) and one Koala isolate (LPCoLN) into all subsequent comparative analyses.

### Genomic variation of *Cpn* clinical isolates

**Figure 1:**
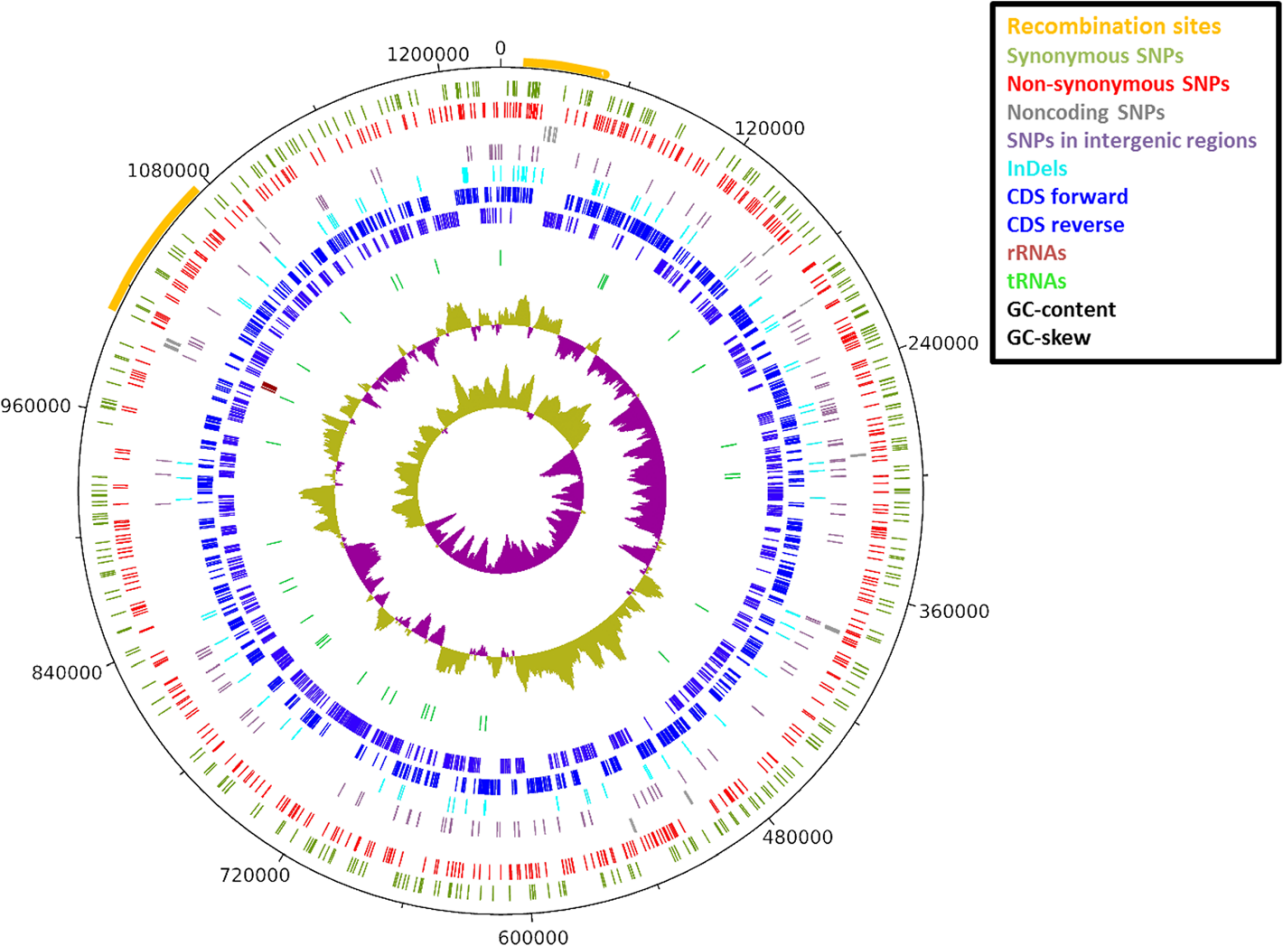
**Variation genome circle.** Genome circle showing the distribution of variations distributed over the *Cpn* genome. Classification of SNPs is based on the CWL029 annotation.

A multiple whole genome alignment of all *Cpn* clinical isolates served as backbone for comparative genomics. We determined a total of 1,363 SNP and 184 InDel loci from the alignment (Figure 1; Additional file 1: Tables S2 and S3 and Additional file 2). These findings indicate very low overall sequence diversity of all sequenced *Cpn* clinical isolates and are in very good agreement with previous studies of selected genomic regions [12-15]. The strategy of whole genome sequencing and comparative assembly, however, revealed a more unequal distribution of variations in the genome. The genes with the highest density of SNPs and InDels mostly encode ‘hypothetical proteins’ and in many cases contain both SNPs and InDels (e.g. CPn_0012, CPn_0010, CPn_0010.1, CPn_0013, CPn_1054) (Additional file 1: Tables S4a and S5a). Variation frequency is generally highest in the intergenic regions (Additional file 1: Tables S4b and S5b).

Taking the annotation of the *Cpn* strain CWL029 as a reference, 47% of all SNP loci (637) have at least one non-synonymous (ns) exchange, 36% (491) are only synonymous (s), 14% (191) are located in intergenic regions and 3% (44) are in noncoding genes. Approximately 75% (137) of the InDels are located in coding sequences. Already in previous studies an unexpectedly high number of non-synonymous SNPs (nsSNPs) were particularly observed when human isolates were compared to non-human isolates [12,13]. It was hypothesized that zoonosis events would temporarily favor positive selection and a higher rate of non-synonymous substitutions, whereas negative selective pressure dominates after successful adaptation. To test this assumption, we calculated the K_a_/K_S_ ratio of non-synonymous versus synonymous substitutions per gene. K_a_/K_S_ ratios of the clinical isolates compared to the animal outgroup DC9 (Additional file 1: Table S6a) were on average higher than the ratios among the clinical isolates (Additional file 1: Table S6b). K_a_/K_S_ ratios > 1 for 54 genes in the comparison to DC9 indicate that these genes were under positive selection during the adaptation to the human host. Comparing only the genomes of clinical isolates, most genes have the expected ratios << 1 of negative selective pressure. Only four genes have K_a_/K_S_ ratios > 1, out of which three genes are ‘putative uncharacterized proteins’ (CPn_0456, CPn_0809 and CPn_1027) while CPn_0598 is a ‘dipeptide transport system permease protein’. However, there are no indications that these genes are pseudogenes.

**Figure 2:**
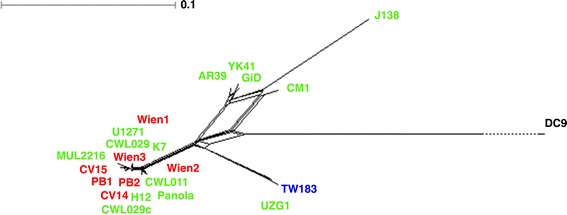
**Whole genome recombination tree.** The split graph obtained from the SNPs between all human *Cpn* isolates and the frog isolate DC9. It contains a split that clearly separates the isolates CM1 and J138 from TW183 and UZG1 and the group consisting of Wien1, U1271, CWL029, K7, MUL2216, Wien3, CV15, PB1, PB2, Wien2, CWL011, CV14, H12, Panola and CWL029c.

Genome recombination has been proposed but could not be shown in *Cpn* so far [11-13]. As our study significantly expands the number of *Cpn* genome sequences, it is now feasible to address the question of recombination. From a multiple genome alignment also containing DC9 as closest representative of non-human isolates, we determined a total number of 4,690 SNP loci. A statistical Phi test and a Bayesian inference approach yielded significant evidence that recombination has occurred between the isolates (p-value < 1e-05). Recombination was observed between human, but not between human and the non-human isolate (Figure 2 and Additional file 3). Testing sliding windows along the genome alignment suggests two regions of recombination, one between the genomic positions 10 Kb and 40 Kb and the second between 1 Mb and 1.07 Mb (Additional file 1: Table S7a and Additional file 4). The areas adjacent to the two regions of recombination did not show evidence for recombination. This suggests an exchange of DNA within these two relatively short genomic regions. Due to the limited number of SNPs the actual recombination sites cannot be determined more precisely. The first region contains 7 pmp-genes of which 6 are annotated as pseudogenes (Additional file 1: Table S7b). The second region is enclosed by the operon of ribosomal RNAs and three ribosomal proteins whereas the genes in between encode various different enzymes (Additional file 1: Table S7c).

### Evolution of the *Cpn* clinical isolates

The newly sequenced genomes of 19 *Cpn* isolates as well as the five publicly available *Cpn* genomes represent the most comprehensive dataset for the reconstruction of the evolutionary history of human pathogenic *Cpn*. We calculated a multiple genome alignment based on the 22 human *Cpn* isolates (Table 1). The two non-human isolates (DC9 and LPCoLN) were integrated as outgroups. From this alignment, we derived a total number of 13,324 SNP loci.

**Figure 3:**
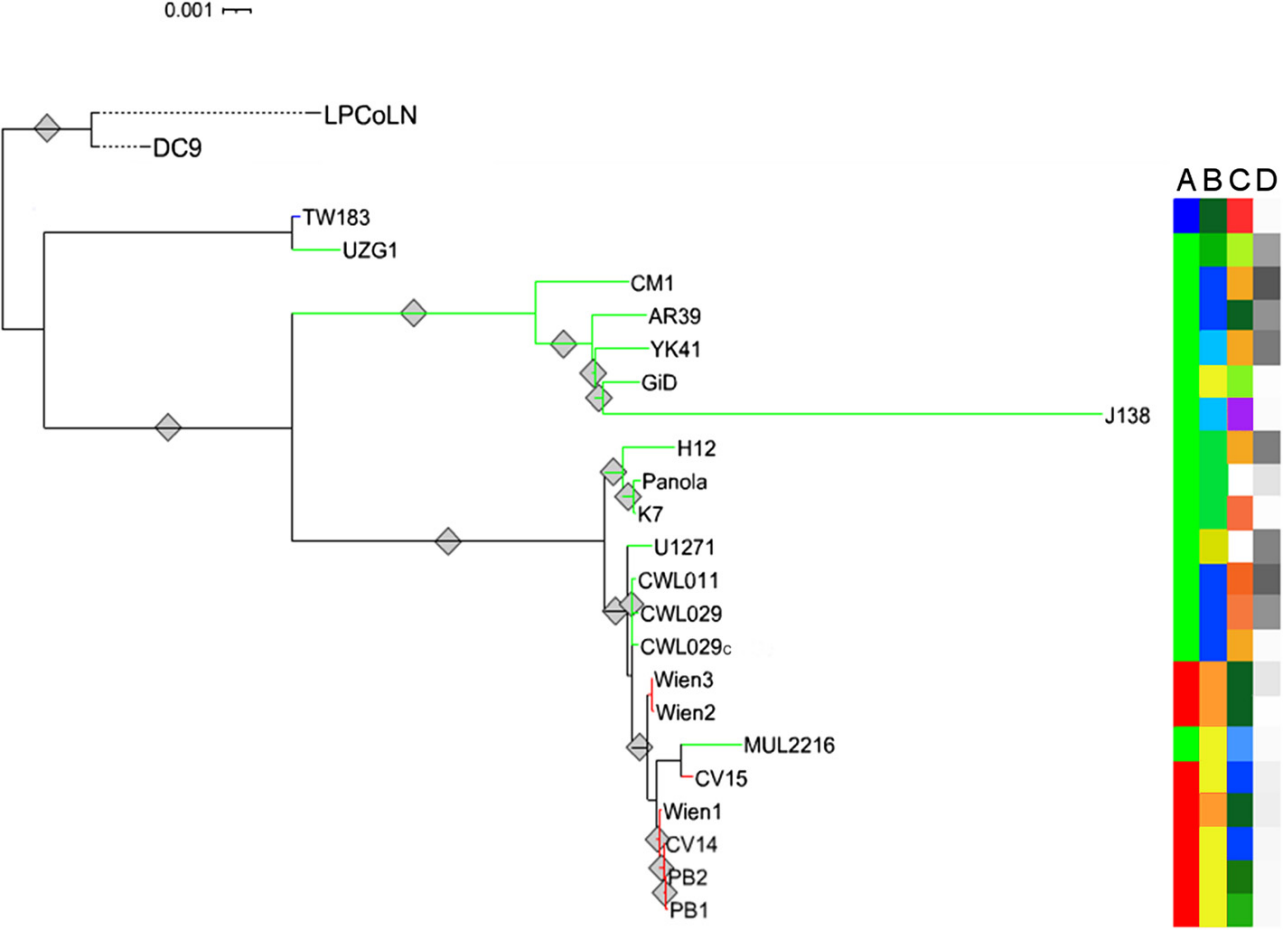
**Single nucleotide polymorphism phylogeny.** Maximum likelihood phylogenetic tree based on SNPs derived from a whole genome alignment. Diamonds on the tree branches indicate bootstrap support > 80. Colors on the right of the tree represent **(A)** the type of tissue: blue: conjunctival, green: respiratory, red: vascular; **(B)** country of origin; dark green: Taiwan, green: Belgium, dark blue: USA, light blue: Japan, yellow: Germany, orange: Austria; **(C)** year of isolation: dark red: 1965, light green: before 1996, orange: before 1992, green: 1983, purple: 1994, white: NA, light red: 1987, dark green: 1998–1999, light blue: 2001, dark blue: 2002, olive: 1998; **(D)** *tyrP* coverage: the gray scale of the *tyrP* coverage indicates the estimated *tyrP* copy number from one copy (white) to two copies (dark gray).

The most stable tree was obtained from all SNP positions, including non-synonymous, synonymous, non-coding and intergenic loci (Figure 3). The tree shows a clear separation between outgroups, DC9 from frog and the koala isolate LPCoLN, and the human isolates. The human isolates split up into three clusters, all supported by high bootstrap values. TW183 and UZG1 form one cluster that branches deeper in the tree than the other two human clusters. The isolates YK41, AR39, GiD and J138 are contained in another cluster together with CM1, which is located in a sister branch within this cluster. The long sub-branch of J138 can be mainly ascribed to a region between genomic positions 1,204,000 and 1,205,000, showing around 110 SNPs that are only present in J138. In J138 no gene is annotated in this region, whereas it is marked as pseudogene CpB1096 in TW138 and contains an annotated gene (CPn_1054) in CWL029. The third cluster contains three sub-groups, all of which are well supported by bootstrap values. The first group consists of H12, Panola and K7; the isolates U1271, CWL011, CWL029 and CWL029c form the second group. The largest sub-group in the branch consists of the isolates Wien2, Wien3, MUL2216, CV15, Wien1, CV14, PB1 and PB2. Inside this sub-group there are again three small groups, the first containing Wien2 and Wien3, the second containing MUL2216 and CV15 and the third consisting of Wien1, CV14, PB1 and PB2. All of these three groups have good bootstrap support. Overall, there is a remarkable congruence between the phylogenetic tree presented in this study and the phylogeny calculated from a set of selected SNP positions reported in an earlier study from Rattei et al. [12], with our new phylogeny providing a much higher resolution of the CWL029 containing cluster. The topology of this tree, based on all SNPs, is also supported by trees based on subsets of SNPs, such as from 31 phylogenetic marker genes (Additional file 5 A) and from 545 genes that represent the chlamydial pan-genome (Additional file 5 B).

**Figure 4:**
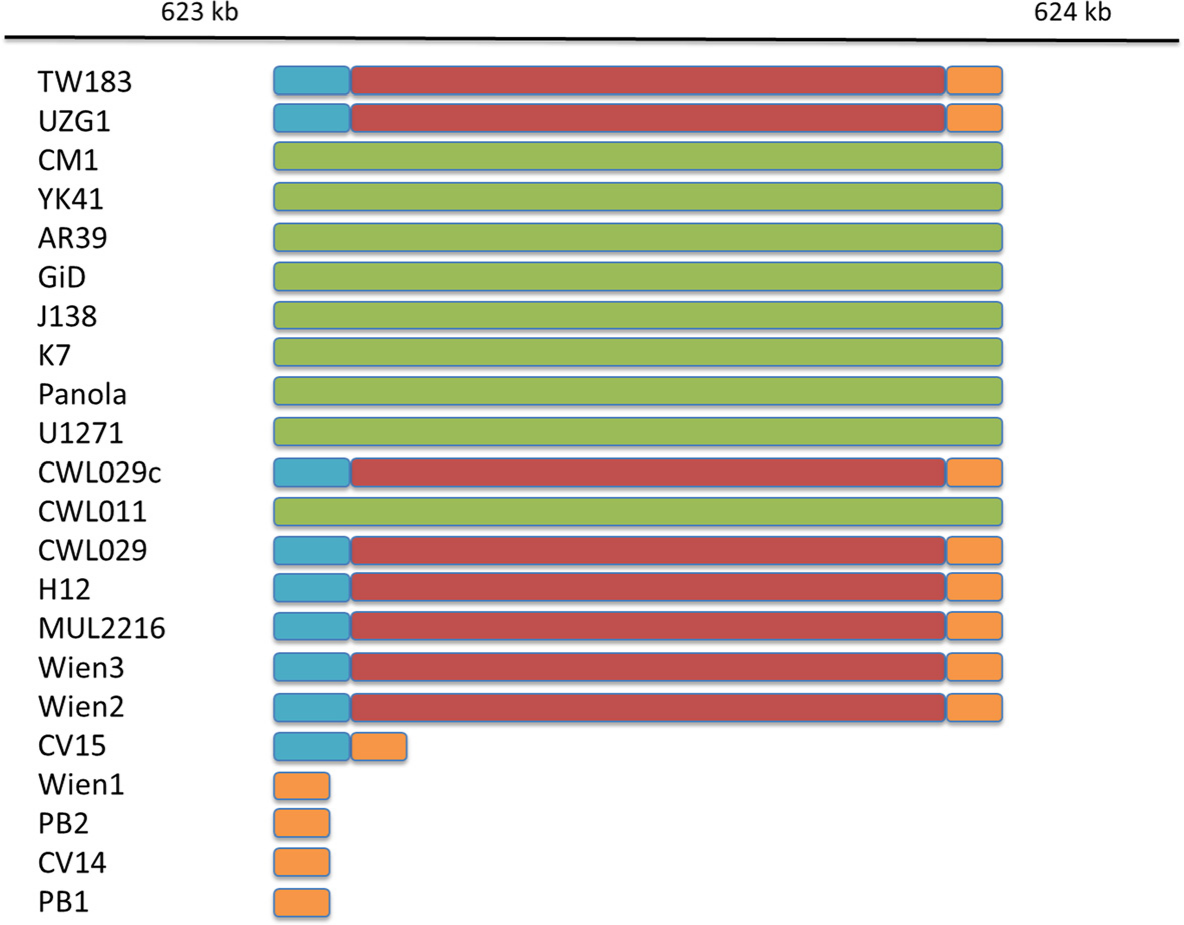
**Whole genome alignment for pmp_20 locus.** A schematic representation of the section containing the pmp_20 locus from the whole genome alignment of all human *Cpn* isolates. Blocks of similar color represent similar sequences in different isolate genomes.

Distances in the new phylogenetic tree are generally in agreement with the number of SNPs between the different isolates but also with the number of InDels between the isolates (Additional file 1: Tables S8 and S9). Whereas the SNPs were used to calculate the phylogenetic tree and therefore are expected to represent the topology, the InDels are independent from the tree bust still show a similar distance pattern. In some cases, not only the numbers of InDels, but also the contained sequences, show a pattern related to the SNP phylogeny. E.g., the five vascular isolates PB1, PB2, CV14, CV15 and Wien1 have a deletion of ~420 nt in the pmp_20 gene, which is not present in any other isolate (Figure 4). In the phylogenetic tree, these five isolates form one sub-group in the branch of vascular isolates.

Although the low number of SNPs limits the phylogenetic reconstruction of specific genomic regions, we calculated phylogenetic trees from SNPs in all 2 Kb regions in the recombination areas determined before. Only a short 2 Kb region between the genomic positions 14 Kb and 16 Kb revealed clear phylogenetic evidence for recombination between the isolates TW183 and UZG1 and the group of vascular isolates. Additional file 4 shows the maximum likelihood phylogenetic tree based on SNPs derived from this 2 Kb region and an additional tree derived from the surrounding region. The isolates TW183 and UZG1 and the vascular isolates are located on the same branch of the tree based on the 2 Kb region, but are clearly separated in the tree of the surrounding region, resembling the topology of the whole-genome based tree.

### Pathogen-related differences with respect to *Cpn* tissue tropism

An earlier study indicated that respiratory strains of *Cpn* clinical isolates contain multiple copies of the *tyrP* gene, whereas vascular strains contain a single copy [8]. In order to determine the *tyrP* copy number in the 18 newly sequenced genomes of human *Cpn* isolates we mapped the sequenced reads specifically against the entire *tyrP* locus of CWL029, containing two *tyrP* gene copies (Additional file 1: Table S10). We determined the *tyrP* copy number in each genome from the average sequencing depth of the *tyrP* locus compared to the upstream and downstream regions. One copy of the *tyrP* gene was observed in all vascular isolates, whereas the respiratory isolates vary between one (GiD, K7, Panola, MUL2216, J138, CWL029c) and two copies (CM1, H12, CWL011, U1271, UZG1, YK41, CWL029, AR39) (Figure 3D, Table 3). The varying *tyrP* copy numbers between several closely related isolates, e.g. in CWL029c and CWL029, might represent intra-strain polymorphism which has been reported previously by Gieffers et al. [8]. Mitchell et al. already described in 2010 that the respiratory isolate J138 and the conjunctival isolate TW183 contain only one *tyrP* copy and questioned the hypothesis that the *tyrP* copy number determines tissue tropism (Mitchell, Hovis, et al. 2010). We have found further non-vascular isolates having only a single *tyrP* gene (CWL029c, K7, GiD). The homogenous presence of a single *tyrP* gene in vascular isolates therefore indicates the close phylogenetic relatedness between these isolates and might contribute to the tissue tropism in the connection with other predisposing genetic factors.

**Table 3:** ***tyrP*** **copy number**

**Isolate**	***tyrP*** **copy number**
CM1	2
CV14	1
CV15	1
CWL011	2
CWL029c	1
DC9	1
GiD	1
H12	2
K7	1
Panola	1
MUL2216	1
PB1	1
PB2	1
U1271	2
UZG1	2
Wien1	1
Wien2	1
Wien3	1
YK41	2
TW183	1
CWL029	2
AR39	2
J138	1

Copy number of the *tyrP* locus for the *Cpn* isolates used in this study. The copy number for the reference genomes was taken from literature, for the newly sequenced genomes it was estimated from the mapping.

The presence of phages in various *Chlamydia* genomes has been shown to be related with chlamydial infection and pathogenicity. Hoestgaard-Jensen et al. could show that ϕCPAR39 expression resulted in T3S disruption and IncA accumulation in the morphological abnormal chlamydial reticulate bodies (RBs) [17]. Searching the newly sequenced genomes yielded no evidence for phage-like sequences in any of the isolates of this study. Neither mapping of the sequenced reads to known *Chlamydia* phages, nor re-assembly of the non-human and non-chlamydia reads, nor the search for phage-related sequences in the InDels, indicated the presence of phages. Thus, we did not find any support that phage-related determinants are among the primary factors for tissue tropism. However, we cannot completely rule out that we missed phages in one or the other isolates as our genome sequencing methodology was not optimized for phage detection [18].

High genomic similarity of isolates from same tissues was found earlier [12,19] and points to specific genotypes that may determine *Cpn* tissue tropism. We therefore assigned attributes characterizing the isolates to the corresponding leaf in the phylogenetic tree (Figure 3A-C). Table 1 presents: the type of tissue from which the isolate was sampled and is used as an identification mark for tropism in this study; the geographical location of the sampling; and the year of sampling. Among these three attributes “tissue” mostly corresponds to the topology of the tree, whereas the others show weak (“location”) or no (“year”) correlation to the genotype. All vascular isolates (Wien2, Wien3, CV15, Wien1, CV14, PB1 and PB2) are located in one, monophyletic and short-branching part of the tree with only one respiratory isolate, MUL2216, in between. It can, however, not be excluded that MUL2216 is potentially a vascular isolate that was just isolated in an early stage of infection. Contrary to that, the attributes “location” and “year” do not show comparable agreement with the genotype. Although vascular isolates were only available from two different countries, the sampling occurred in different years in which respiratory isolates also were collected. The remarkable agreement between the clusters formed by the attribute “tissue” and the topology of the tree suggest genotypic factors to be involved in the *Cpn* tissue tropism.

Since the phylogenetic tree showed a clear separation of respiratory and conjunctival from vascular isolates we determined the SNP positions distinguishing the two groups. In total, nine locations that are characteristic for the type of the isolate (respiratory/conjunctival or vascular) were found (Table 4). These SNPs are all non-synonymous and located in genes involved in RB to EB transition, inclusion membrane formation, bacterial stress response and metabolism. For several of the genes, biological functions have already been elucidated that could have a direct impact on implementation of *Cpn* infections under the prerequisite of different environmental conditions of the lung and the vasculature. For example, CPn_0289 regulates the uptake of glutamate that has been shown to stimulate growth of *Cpn* but not *C. trachomatis* [20]. The glutamyl tRNA reductase (CPn_0714) mediates the metabolism of phenylalanine that is involved in the extracellular protein synthesis of the transcription termination factor Rho and the Nudix phosphohydrolase in *C. trachomatis*, thereby mitigating cellular stress responses [21]. Special focus should be given to the regulatory and stress response genes CPn_0081 (RNA polymerase beta) and CPn_0793 (pp2c phosphatase) to look for bacterial adaption mechanisms in different microenvironments. In terms of pathogenicity and intracellular host-pathogen interactions, further investigations on the transcriptional relevance of the nsSNPs in the *incC* gene (CPn_0292) and the acyl-carrier UDP-GlcNAc O-acyltransferase (Cpn_0650) are needed. To substantiate our findings, we checked these characteristic SNPs in additional two respiratory isolates from the US (W5 and W6) and one additional respiratory (MUL2076) and vascular (CV6) isolate from Germany. We sequenced the respective regions harboring the characteristic sSNPs in CPn_0081, CPn_0650 and CPn_0920, the isolates grouped within the newly defined vascular and respiratory cluster, respectively (Additional file 1: Table S11).

**Table 4:** **Tissue-specific SNPs**

**Position**	**Locus_tag**	**Description**	**Clinical isolates from the respiratory tract**														**Clinical isolates from blood monocytes and artery vessels**							**Clinical isolate from conjunctiva**
			**CM1**	**CWL011**	**CWL029**	**GiD**	**H12**	**K7**	**MUL2216**	**U1271**	**UZG1**	**YK41**	**J138**	**AR39**	**Panola**	**CWL029c**	**CV14**	**CV15**	**PB1**	**PB2**	**Wien1**	**Wien2**	**Wien3**	**TW183**
95,992	CPn_0081	RNA Polymerase Beta	G	G	G	G	G	G	G	G	G	G	G	G	G	G	T	G	T	T	T	G	G	G
326,721	CPn_0289	Neutral Amino Acid (Glutamate) Transporter	G	G	G	G	G	G	T	G	G	G	G	G	G	G	T	T	T	T	T	T	T	G
329,607	CPn_0292	Inclusion Membrane Protein C	G	G	G	G	G	G	T	G	G	G	G	G	G	G	T	T	T	T	T	T	T	G
576,846	CPn_0496	CT391 hypothetical protein	C	C	C	C	C	C	A	C	C	C	C	C	C	C	A	A	A	A	A	A	A	C
733,006	CPn_0650	Acyl-Carrier UDP-GlcNAc O-Acyltransferase	C	C	C	C	C	C	C	C	C	C	C	C	C	C	G	G	G	G	G	C	C	C
800,395	CPn_0714	Glutamyl tRNA Reductase	G	G	G	G	G	G	G	G	G	G	G	G	G	G	T	T	G	T	T	T	T	G
896,637	CPn_0793	sigma regulatory family protein - PP2C phosphatase (RsbW antagonist)	G	G	G	G	G	G	T	G	G	G	G	G	G	G	T	T	T	G	T	T	T	G
1,050,652	CPn_0920	Sulfite Synthesis/biphosphate phosphatase	C	C	C	C	C	C	C	C	C	C	C	C	C	C	A	A	A	A	A	C	C	C
1,156,117	CPn_1006	CT849 hypothetical protein	T	T	T	T	T	T	C	T	T	T	T	T	T	T	C	C	C	C	C	C	C	T

SNP loci that distinguish respiratory and vascular isolates with the corresponding genes in CWL029 and their genotype in the different isolates. All SNPs are non-synonymous.

## Conclusions

While acute *Cpn* infections of the upper and lower respiratory tract are frequently seen in clinics and seem to emerge in small endemic episodes, the relevance of *Cpn* infections in the pathogenesis of atherosclerotic vascular diseases is less clear. Although experimental models support the hypothesis that *Cpn* contributes to the development of atherosclerotic lesions by direct or indirect mechanisms [22], clinical evidence is still missing. We were interested in the genetic background of clinical isolates from *Cpn* that were derived from different anatomical origins. Selection of isolates was motivated by the availability of re-cultivable strain preparations, clear documentation of the isolation source and the endeavor to obtain as large geographical distribution as possible. The only vascular strain A03 [23] from the US we had access to was severely contaminated with *Mycoplasma* and could not be re-cultivated. Previous studies determined the genetic variation in *Cpn* from selected SNPs [12] or gene sequences [13] rather than complete genomes due to the high sequencing costs at that time, which are particularly exacerbated by an unavoidable proportion of human DNA in chlamydial samples. The recent progress in DNA sequencing techniques makes it feasible to sequence a sufficient amount of DNA to reconstruct entire genomes at low costs. In this study, we therefore applied the Illumina technique to 19 *Cpn* isolates. Independent of our study, Harris et al. recently pursued a similar approach to sequence 36 *C. trachomatis* genomes [10], also based on next-generation sequencing. Due to the shortness of the reads (36nt, 54nt and 76 nt) and for some isolates the limited amount of sequence data, neither mapping to the CWL029 genome as a reference, nor de novo assembly allowed us to assemble complete genomes. To overcome this limitation, and in difference to the manual scaffolding used for *C. trachomatis* [10], we applied a comparative assembly approach. It automatically combines mapping against a closely related reference genome and de novo assembly of regions that cannot be mapped to the reference. Comparative assembly works the better the closer the reference genome fits the target genome. Therefore, we selected the closest reference genome for each of our isolates individually. This special approach allowed us to assemble the complete genomes of six out of 19 isolates as well as genomes consisting of less than 10 contigs for a further nine isolates. The number of contigs for the four remaining isolates ranges between 13 and 147. The quality of these assemblies was verified by comparison with previous genotyping, which indicated a very high accuracy of the obtained genome sequences. High quality of the detected variants was ensured by requiring a sufficient quality (Phred score > = 20) and read coverage (> = 3). A comparison to the genome of the *Cpn* reference strain CWL029 yielded 6 SNP loci as well as 6 InDels, where all isolates have the same genotype except for CWL029 (Additional file 1: Tables S2 and S3). These positions represent likely errors in the CWL029 reference genome and were not observed in the CWL029-labstrain CWL029c formerly obtained from ATCC. Our findings were additionally validated by Sanger sequencing of the respective genomic loci.

We detected very low sequence diversity of the human *Cpn* isolates and a much higher evolutionary distance of the animal isolates confirming results from previous studies [12,19]. However, the unmatched number of 24 complete *Cpn* genome sequences allowed us to investigate the evolutionary relationships between *Cpn* isolates at a novel resolution level. High bootstrap support in most parts of the phylogenetic tree underpins the statistical significance of our results. We found significant evidence of recombination between human *Cpn* isolates and identified a region of 2 Kb around the putative IncA pseudogene Cpn_0010.1 that recombined between the CWL029 group, TW183 and UZG1. Thus, *Cpn* shows in principle similar evolutionary mechanisms as *C. trachomatis,* in which recombination occurs to an even greater extent [10]*.* Also, considering the overall number of SNPs as well as the SNP distribution, we found that human *Cpn* isolates show much less variation than human *C. trachomatis* isolates. Earlier studies already pointed to an unexpectedly high nsSNP/sSNP ratio, suggesting that also sSNPs underlie selection in *Cpn* genome evolution [12]. Our genome-wide dataset allowed an unbiased, gene-wise determination of the selective pressure. Remarkably, positive selective pressure (K_a_K_s_ ratio > 1) was found for four genes and suggests a relationship of these genes to host pathogenicity which requires fast adaptation. However, only for one of them a putative function (Dipeptide transport system permease protein) could be predicted. The variations in plasticity zones suggested by earlier studies [15,19] were confirmed by our data; however, these are not in regions of particular high variability in our multi-genome alignments. Although we recovered other candidates for highly variable regions in the *Cpn* genomes, the existence of plasticity zones as observed in *C. trachomatis* [15] remains questionable.

Only a few studies directly addressed the question of genetic factors that are related to tissue tropism in *Cpn* infections to date. The most extensive showed minor polymorphisms present in the variable domain 4 (VD4) region of the outer-membrane protein-A (*ompA*) gene and the intergenic region between the *ygeD* and uridine kinase (*ygeD–urk*) of *Cpn* infecting human atherosclerotic carotid plaques [9]. The polymorphic outer membrane protein (*pmp*) genes have been suggested previously as a marker for intra-species variation of *Cpn*, differentiating isolates by the size of their PCR product [13]. We observed not only a high number of recombination in the *pmp* genes in general but also a deletion of ~420 nt in *pmp20* in five of the vascular isolates, suggesting a central functional role of this highly variable region in chlamydial diseases [11]. For *Cpn* it was shown that *pmp20* acts as adhesin, is required for efficient intracellular infection and promotes NFκB- mediated pro- inflammatory signaling cascades in vascular endothelial cells [24,25], linking *pmp20* to the vascular pathogenesis of *Chlamydia*.

Our data indicate that multiple copies of *tyrP* are not a prerequisite for respiratory isolates, which is in contrast to previous publications [8]. On the other hand, all vascular isolates harbored only one single copy that could be a consequence of their close relatedness. A functional relevance, however, might exist in the context of persistence development. In this regard the isolate MUL2216, that has been isolated from bronchoalveolar-lavage fluid (BAL) of a 43 year old male patient suffering from COPD and chronic, non-productive cough, is of particular interest, as it expresses five out of the nine nsSNPs classified as “vascular genotype” and contains only one *tyrP* copy. To test the hypothesis whether polyclonal infections occur *in vivo*, it would require to subsequently genotype *Cpn* from DNA samples derived from respiratory samples and whole blood within short time after respiratory resolution of the respiratory symptoms [26] like it was done before for *C. trachomatis* isolates from clinical swabs [27]. A particular focus should be given in further studies to the non-respiratory, non-vascular human pathogenic isolates. Dean et al. could show in a trachoma-endemic area that infections with *Cpn* were significantly associated with severe conjunctival inflammation overlapping with the clinical appearance of *C. trachomatis* induced trachoma [28]. However, only few cases of *Cpn* induced conjunctivitis were reported so far [29] so that re-cultivable isolates were not adequately represented in our study.

Taken together, our data highlight for the first time genetic differences of clinical *Cpn* isolates that are connected to the anatomical origin of cultivation. With novel technologies for genetic modification of *Chlamydia* at hand, a detailed functional characterization of the different clinical isolates with respect to growth, extracellular survival, stress responsiveness and persistence induction is required. In addition, a clinical evaluation of atherosclerotic patients that is not based on the presence of anti-chlamydial antibodies but on the genetic background of the respective clinical isolate could foster our understanding of the pathophysiological relevance of *Cpn* infections in these patients and help to optimize diagnostic tools for the identification of vascular infections.

## Methods

### Selection of the clinical samples, sample preparation and sequencing

Table 1 lists all clinical *Cpn* isolates that were used in this study. In total 24 isolates were selected from a pool of 38 isolates that were used in the study from Rattei et al. [12] according to three stringent characteristics: 1) highest variety in geographical origin; 2) highest variety in anatomical origin; and 3) possibility to purely cultivate the pathogen. Some isolates that would have met the criteria like AL-1, T-45 (respiratory tract, Umea/Sweden) or A03 (coronary artery, Louisville/USA) had to be excluded because the isolate could not be re-cultivated or was contaminated with other bacteria, respectively. The CWL029 isolate that was propagated over several cultivation passages is designated CWL029c. *Chlamydia* were cultivated on HEp-2 cells as described previously [30], purified by centrifugation on 30% Urografin (Schering, Berlin, Germany) and chlamydial DNA was extracted using the Nucleo Spin Tissue kit (MacheryNagel, Dueren, Germany) and Proteinase K digestion. Clinical isolates were cultivated in strict separation to avoid cross-contamination. Sequencing was performed on the Illumina Genome Analyzer II using 36 bp, 54 bp or 76 bp paired-end runs.

### Filtering and mapping of the reads

We determined the optimal criteria for minimal read length, minimal quality and requirement of mate-pair reads for each isolate based on the different dataset characteristics using FastQC v0.10.0 [31]. Length and quality criteria were used to filter the reads using fastq_quality_trimmer from the FASTX Toolkit 0.0.13 [32]. Reads without a mate were filtered out using an in-house python script. Reads retained from the filtering were mapped against a combined reference genome consisting of *Cpn CWL029* (NC_000922.1) and human (NCBI Build 37.3, accession GCF_000001405.17) using BWA (version 0.5.9) [33] and samtools 0.1.12a [34]. Reads mapping to human were identified using samtools (command “samtools view” with additional parameter “-f 0x0002” for reads mapped in a proper pair) and subtracted from the filtered reads using an in-house python script.

### Check for polyclonal isolates

The mapping was used to derive a consensus pileup file (command “samtools pileup -c”). We then performed SNP calling and filtered out error-prone variant calls having a low SNP quality according to the samtools documentation (command “samtools.pl varFilter < consensus pileup file > | awk '$6 > = 20'”). The SNP calling gives either the nucleotide of the homozygous SNP or an IUPAC ambiguity code if it is a heterozygous SNP. A significant amount of heterozygous SNPs would indicate the presence of polyclonal isolates. We determined the numbers of homozygous and heterozygous SNPs for each isolate.

### Determination of *tyrP* copy number

We determined the *tyrP* copy number from the sequencing depth of the *tyrP* locus (CWL029-based genomic position 1,111,812 to 1,115,418) relative to the 3 Kb upstream and 3 Kb downstream region for each isolate in the mapping against the reference CWL029. Therefore, we measured the average coverage in each of these three regions based on the mapping described above. The coverage of the *tyrP* locus compared to the neighboring regions indicates the copy number of the *tyrP* gene: if the coverage of the locus and the surrounding regions are similar, the isolate most likely contains two copies of the *tyrP* gene; a coverage of the *tyrP* locus which is half as high as the coverage of the surrounding region points to only one copy of the *tyrP* gene in the isolate. Three or more copies of the *tyrP* gene would be indicated by coverage of the *tyrP* locus that is significantly higher than the coverage of the surrounding region.

### Genome assembly and annotation

We used the comparative assembler AMOScmp-shortReads-alignmentTrimmed from the AMOS framework 3.1.0 [35] with default parameters for the combined mapping and de novo assembly of the remaining non-human reads. The reference genome with the smallest distance in the phylogenetic tree presented in the study from Rattei et al. [12], was used as reference in the comparative assembly for the corresponding isolate. The non-human reads were mapped to the newly assembled genomes using BWA [33] (default parameters) and the coverage per nucleotide was determined with samtools [34]. The read coverage per isolate was visualized using R [36].

The assembled genomes were annotated using a house-internal workflow that integrates *ab initio* predictions from Glimmer [37], Genemark [38], Prodigal [39] and Critica [40] with homology information derived from a BLAST [41] search against NCBI NR. Noncoding RNAs were identified by tRNAscanSE [42], RNAmmer [43], and the Rfam [44] database and functional annotation of coding sequences was based on interproScan [45] and homology searches against the databases Swissprot and trEMBL [46].

### Multiple genome alignment and variant detection

A multiple genome alignment of the assembled contigs of the 18 human isolates as well as the publicly available genomes of human isolates was calculated using the progressiveMauve algorithm implemented in the program Mauve (version 2.3.1) [47] and SNPs were identified by Mauve. InDels were inferred from gaps in the Mauve alignment using in-house perl scripts. Contig boundaries (defined as first and last 10 nucleotides in a contig) were removed to prevent spurious SNPs and InDels.

Polymorphic regions were identified using a sliding window approach looking for regions with at least 5% variation in a 100-nucleotide window. Customized python scripts were used to compare SNP positions and variants identified in the current study to the data of the previous study by Rattei et al. [12]. SNPs were classified as synonymous, non-synonymous and noncoding based on the CWL029 GenBank annotation and SNPs, InDels and the CWL029 GenBank annotation were visualized in a genome plot using DNAPlotter [48].

For phylogenetic reconstruction, we calculated (as described above) a multiple genome alignment based on the 18 human isolates and the four publicly available genomes of *Cpn* clinical isolates. The two non-human isolates DC9 and the publicly available *Cpn* koala strain LPCoLN were included as outgroups. SNPs were derived from the multiple genome alignment as described above and used as input for phylogenetic analysis.

A third multiple genome alignment, (calculated as described above) including all newly sequenced isolates and the publicly available *Cpn* isolates except the koala isolate LPCoLN, accounts for the relatively high dissimilarity of LPCoLN to both DC9 and the human isolates. This third multiple genome alignment was the basis for recombination and K_a_K_S_ analyses.

### Phylogeny

A maximum likelihood phylogenetic tree based on an alignment of the concatenated SNP variants of each isolate was calculated using RaxML [49] v7.2.6 (parameters “-m GTRGAMMA -x 12345 -N 1000 -f a”). The tree topology was manually compared to phylogenetic trees from bayesian inference calculated by MrBayes [50] (v3.1.2, parameters: “lset parsmodel = yes; mcmc ngen = 100000 samplefreq = 100; sump burnin = 250; sumt burnin = 250;”). The maximum likelihood phylogenetic tree was visualized using iTOL [51].

AMPHORA2 [52] was applied to the reference genome CWL029 to identify the coordinates of phylogenetic marker genes within the genome. SNP variants within these genes were concatenated and a maximum likelihood phylogenetic tree was calculated as described above. A third maximum likelihood tree was calculated from SNPs located in the 545 genes that are members of the *Chlamydiae* non-supervised orthologous groups ChlaNOGs in the Eggnog-4.0 database [53] and represent the chlamydial pan-genome.

### Determination of the Ka/Ks ratio

For calculating the ratio of non-synonymous (K_a_) and synonymous substitution rates (K_S_), orthologous proteins were identified using Mauve. Alignments of protein-coding DNA sequences for all possible ortholog-pairs were created using ParaAT [54]. The K_a_/K_S_ ratio for each ortholog-pair was calculated based on the alignment using the program KaKs_Calculator_2.0 [55] with default parameters.

### Analysis of genomic recombination

Recombination between the different isolates was analyzed using the Phi test for recombination implemented in the tool SplitsTree4 [56]. Therefore, the concatenated SNP variants of each isolate were aligned and a neighbor-joining phylogenetic tree was calculated using the program readAL [57]. The phylogenetic tree was used as input for SplitsTree4. In order to identify recombination hotspots, the analysis was repeated using fragments of the genome. Additionally, ClonalFrame [58], another program to test for recombination, was applied to the multiple genome alignment produced by MAUVE.

## Additional files

Additional file 1:
**Supplementary tables.** Excel table containing all Tables S1-S11 that are referenced in the manuscript as separate worksheets. Additional file 2:
**Genome coverage per genome.** PDF image showing the genome coverage per isolate. Additional file 3:
**ClonalFrame consensus phylogenetic tree.** PNG image showing the consensus phylogenetic tree reconstructed by ClonalFrame. Additional file 4:
**Phylogenetic trees for recombined region CPn_0010.1.** PNG image showing maximum likelihood phylogenetic trees for recombination-site 1; A: based on concatenated SNPs from genomic position 1 to 14 Kb and 16 Kb to 30 Kb; B: based on SNPs between genomic positions 14 Kb to 16 Kb. Additional file 5:
**Single nucleotide polymorphism phylogeny based on specific gene sets.** PDF image showing maximum likelihood phylogenetic trees based on SNPs located in (A) 31 phylogenetic marker genes, (B) 545 genes that occur in all chlamydia.
