# Supplementary figures and images for: Genomic factors related to tissue tropism in Chlamydia pneumoniae infection
Source: BMC Genomics. 2015 Apr 7;16(1):268. doi: 10.1186/s12864-015-1377-8 (PMC4489044; doi:10.1186/s12864-015-1377-8)

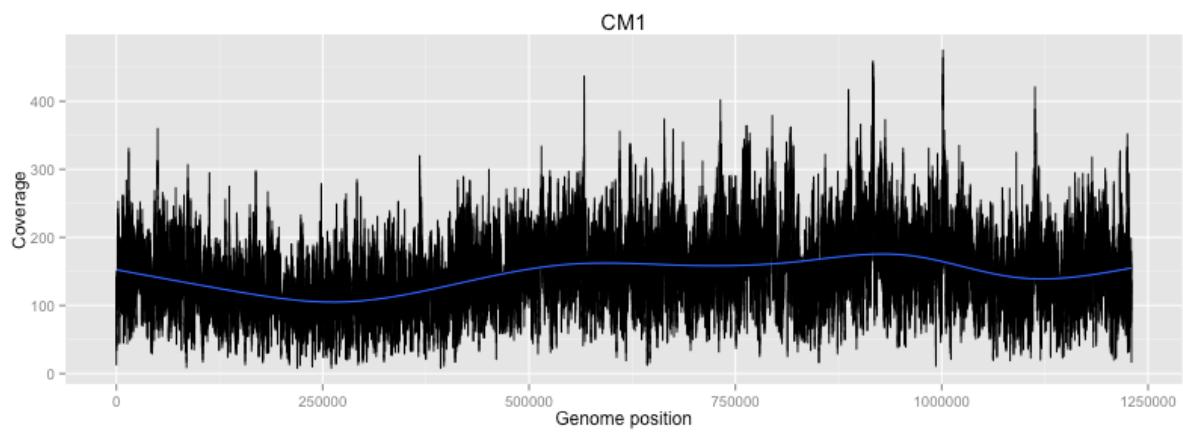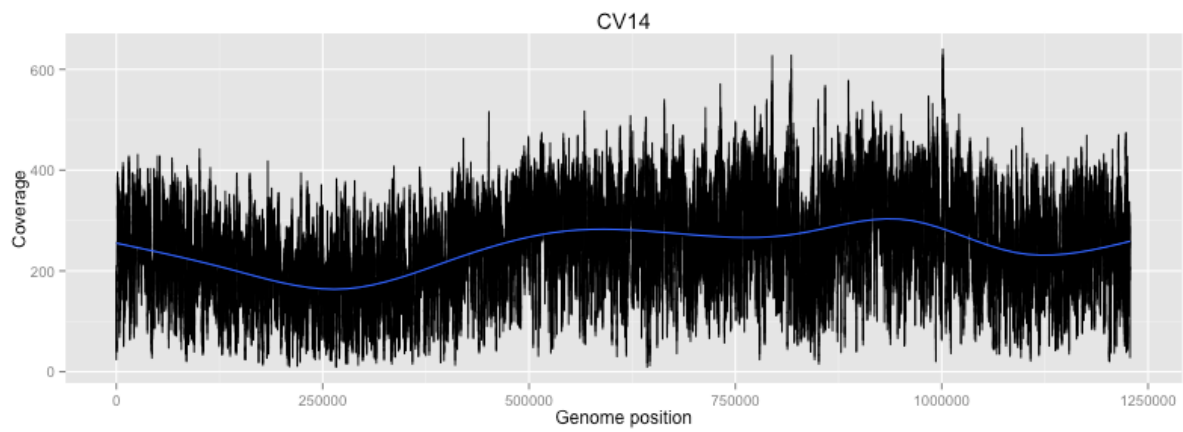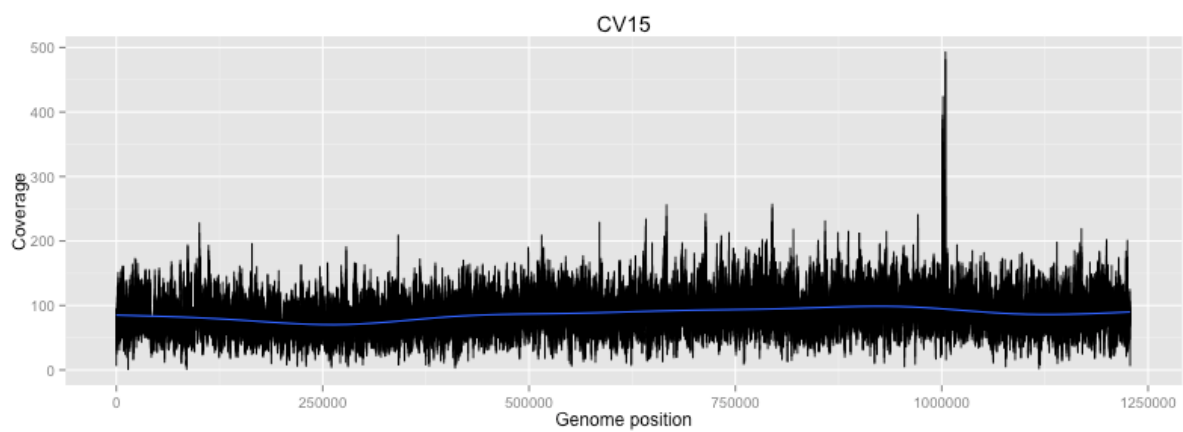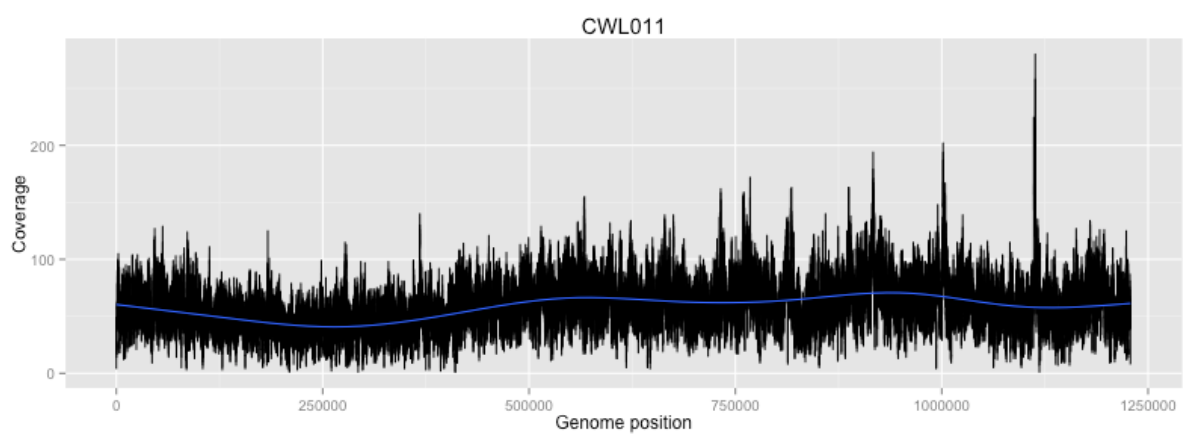

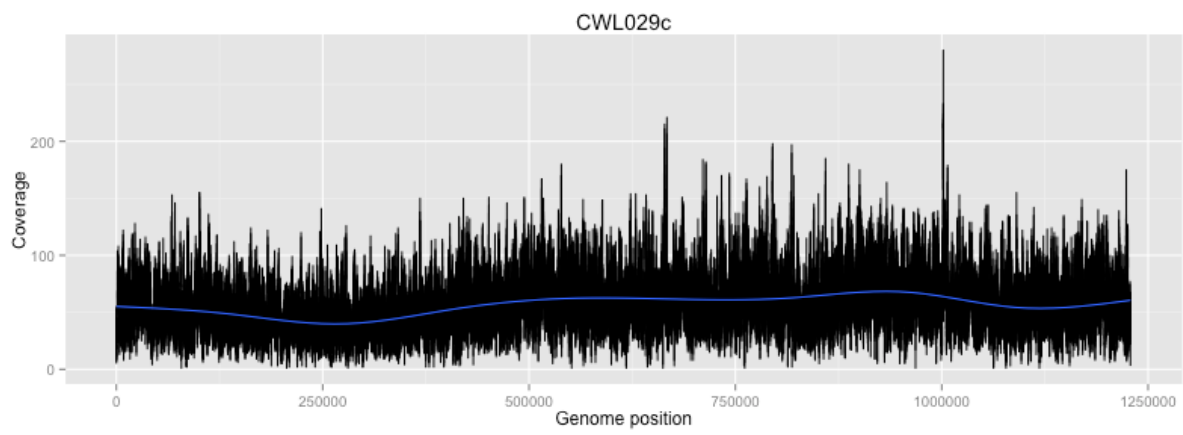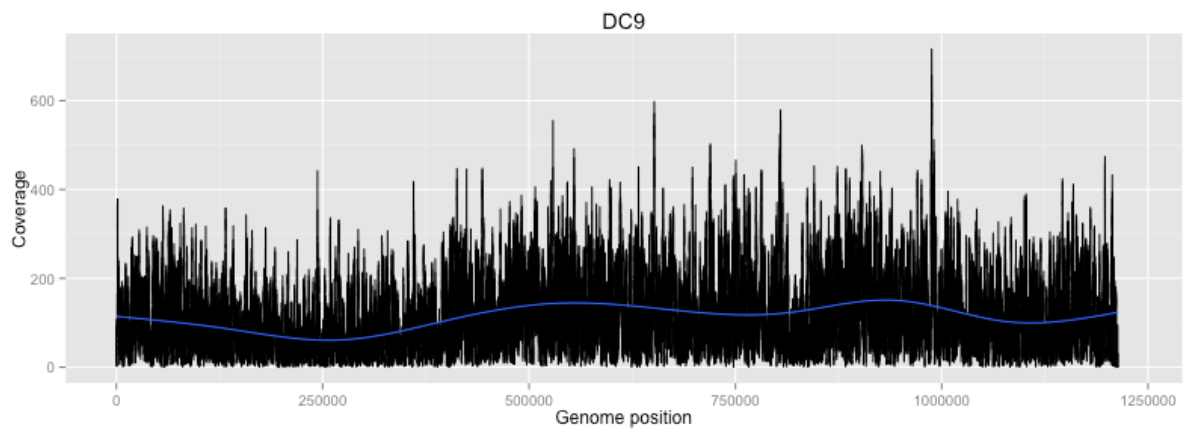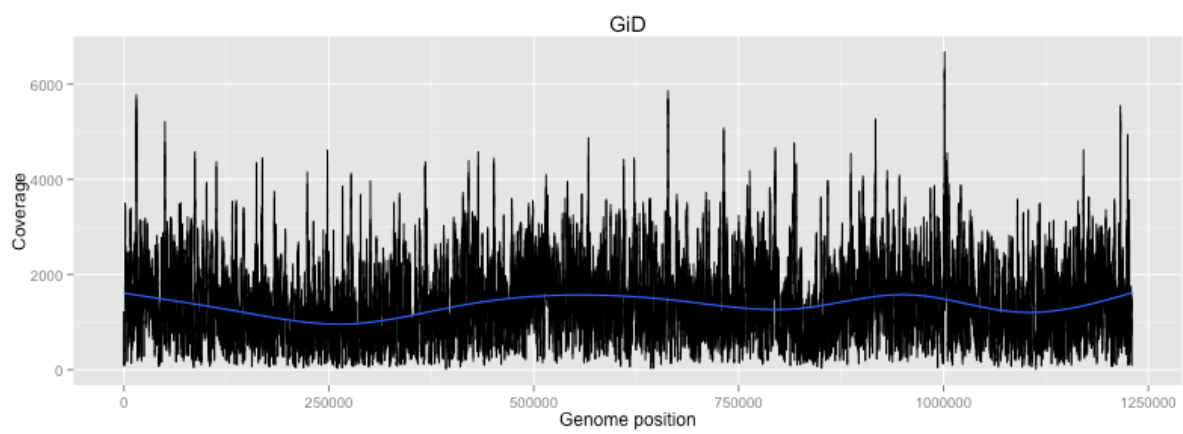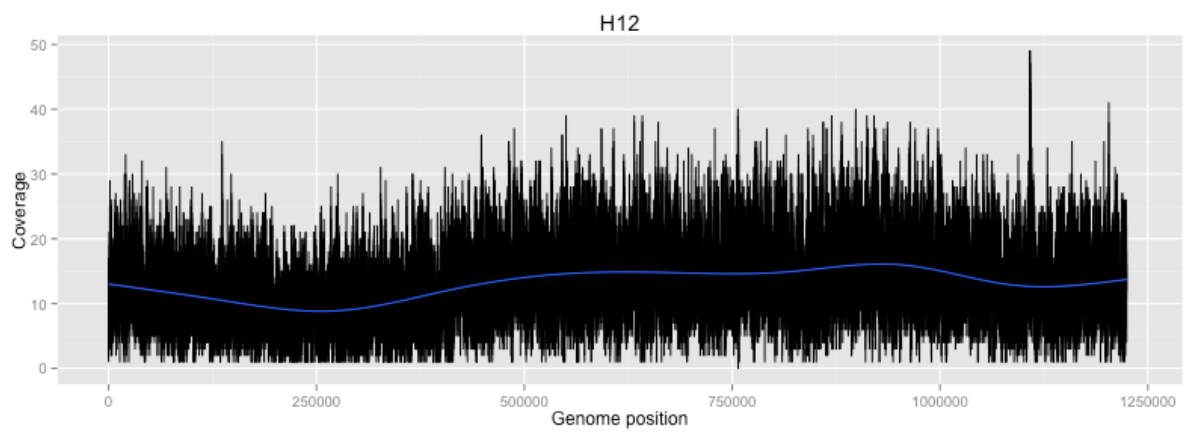

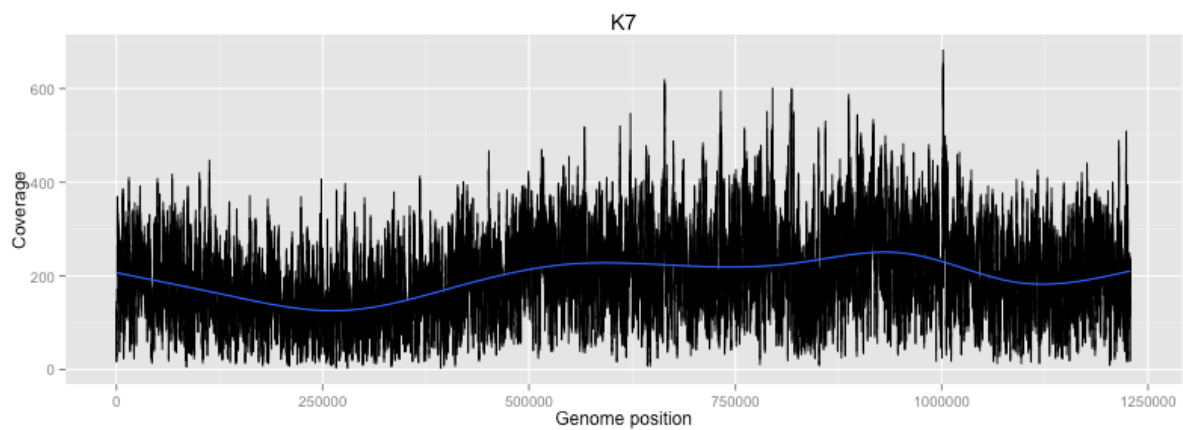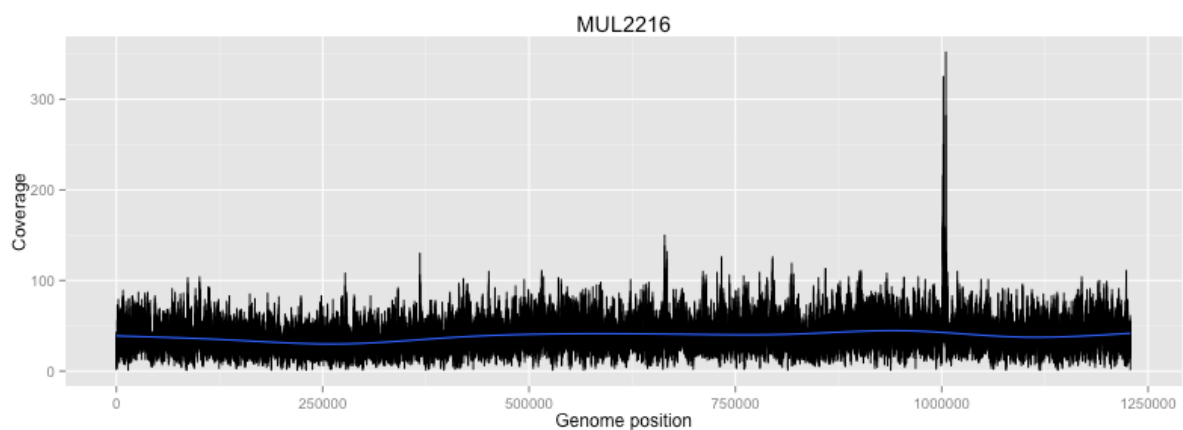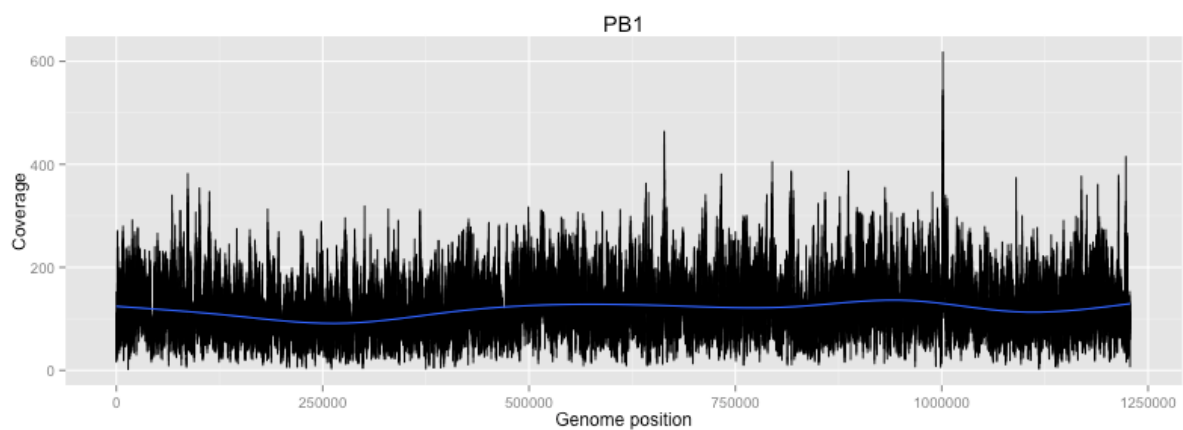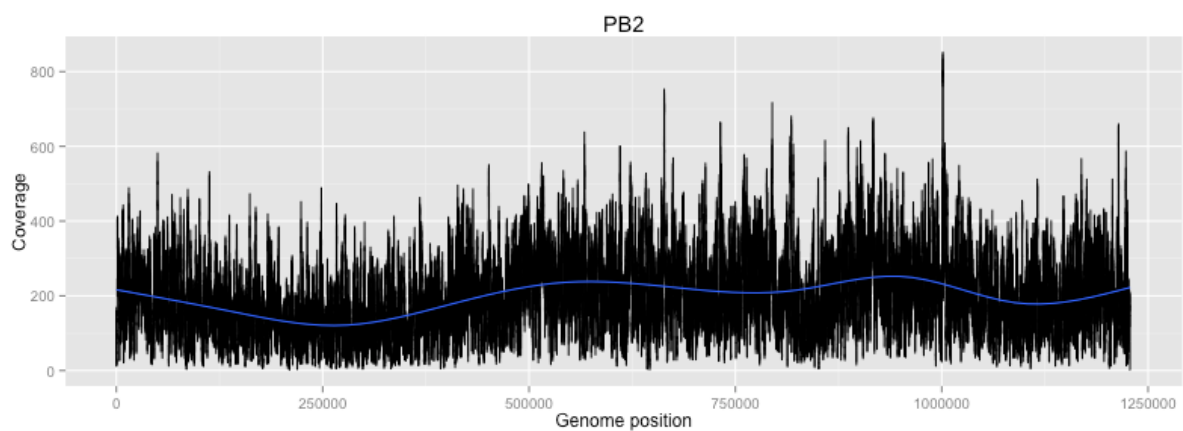

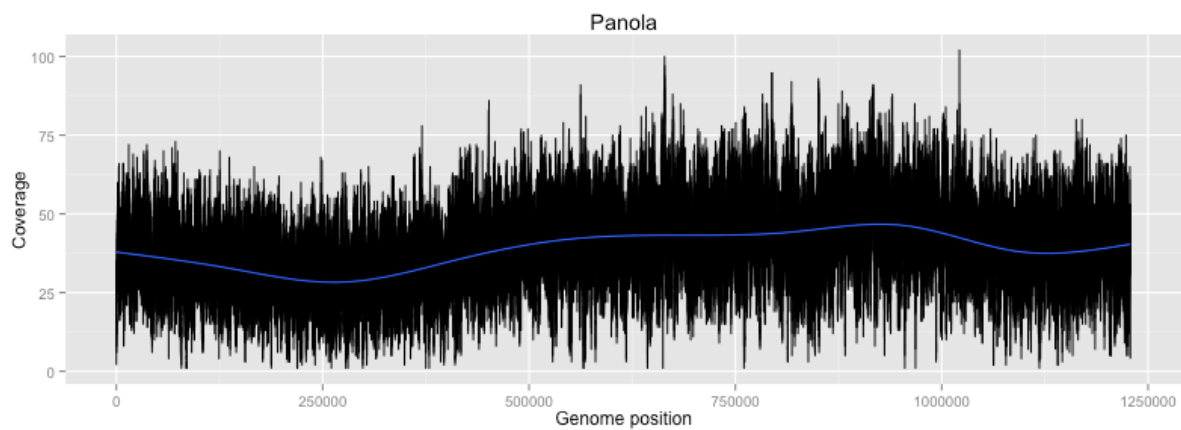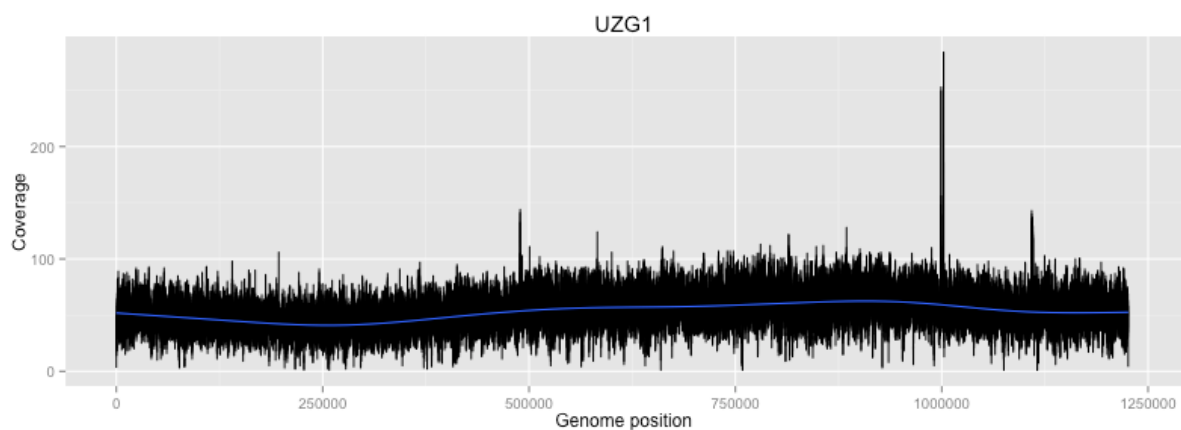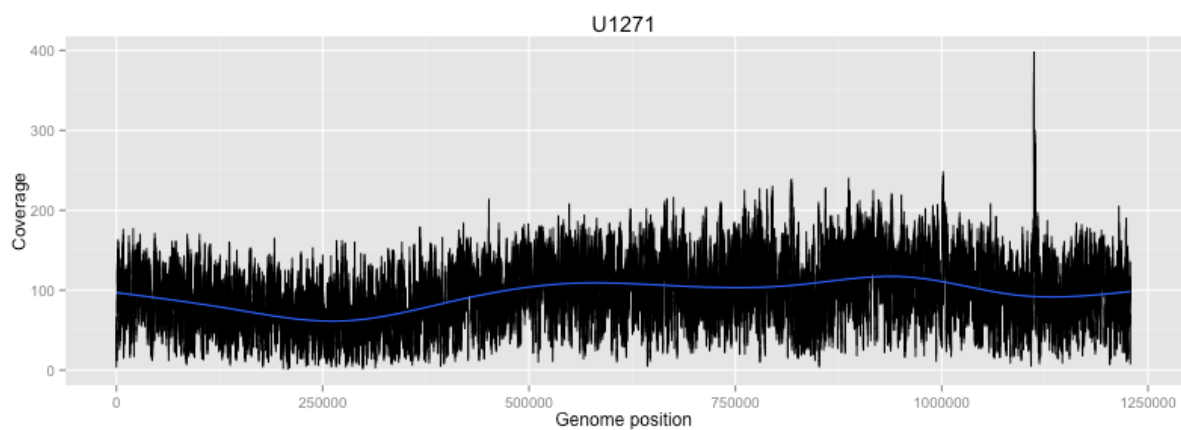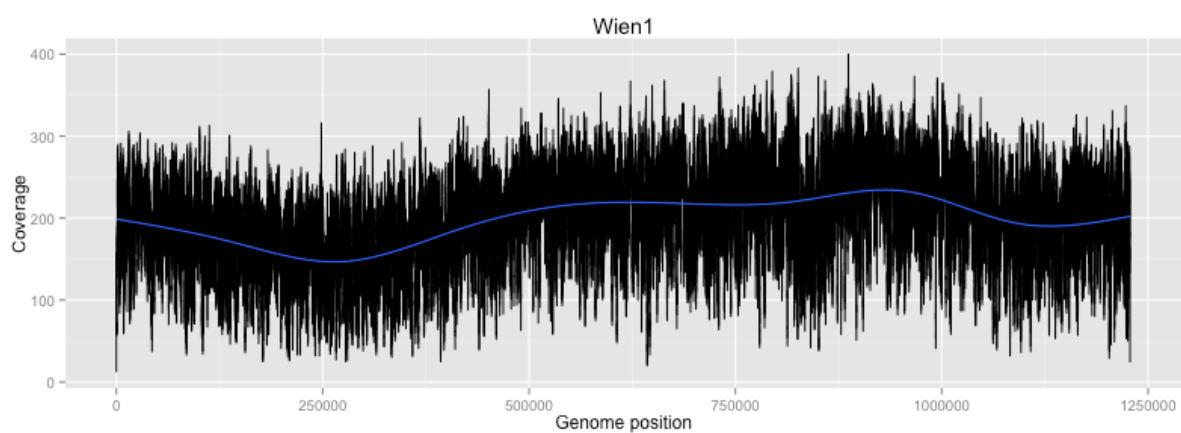

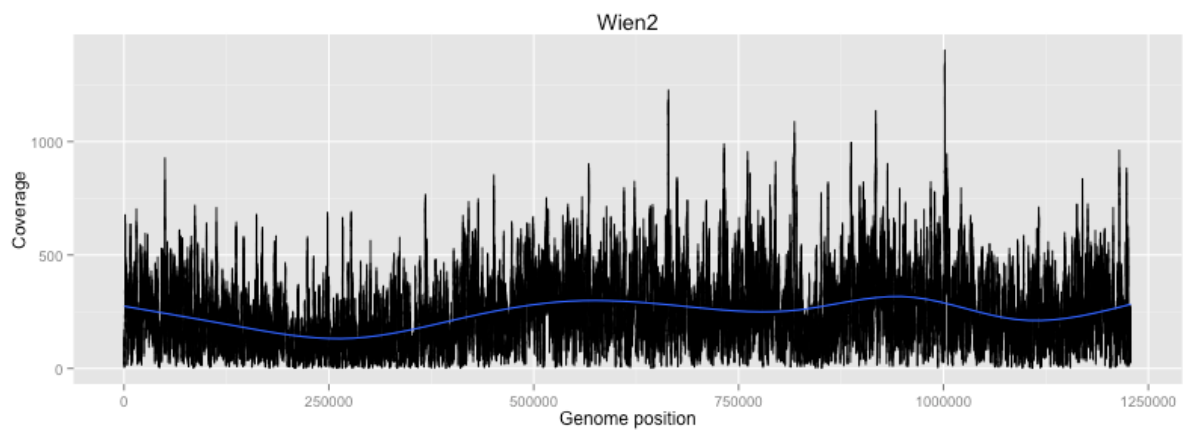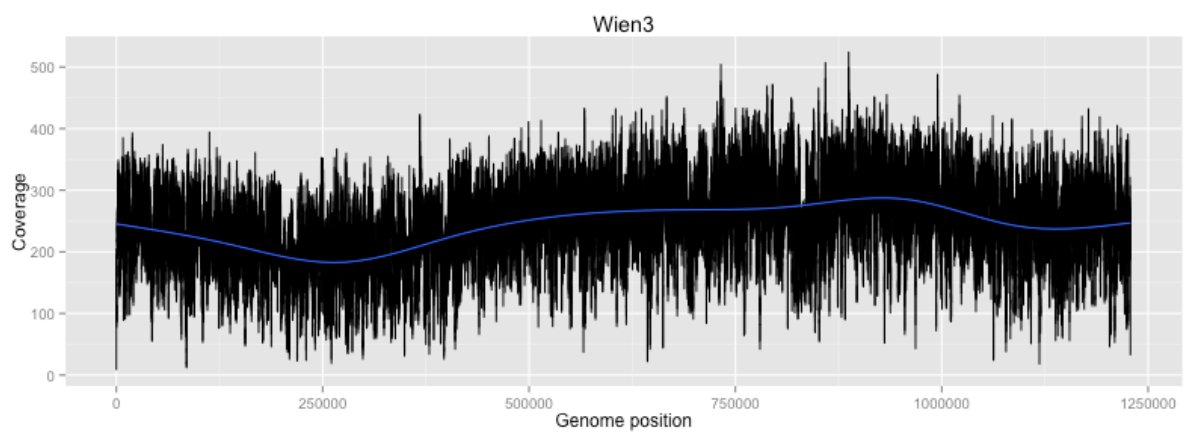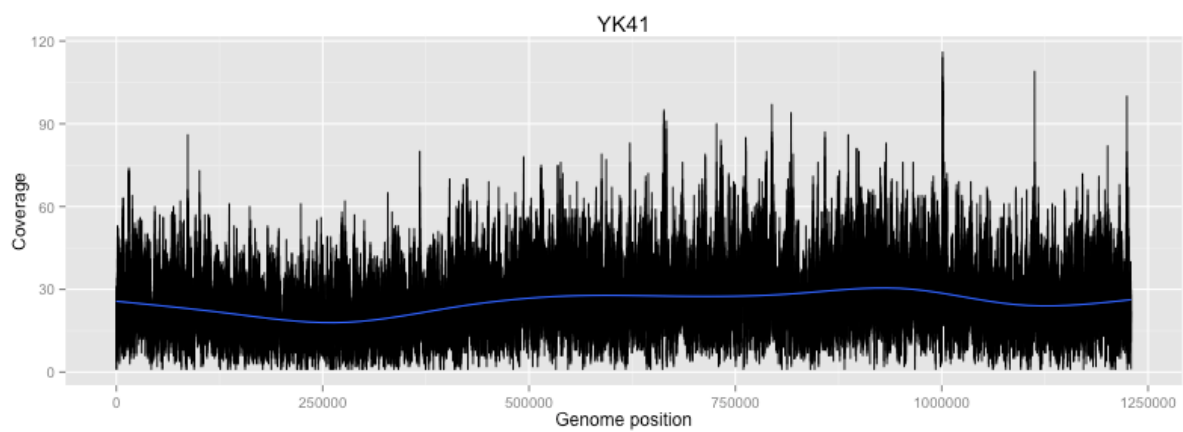

Supplement: Additional file 2: — Genome coverage per genome. PDF image showing the genome coverage per isolate. [file 12864_2015_1377_MOESM2_ESM.pdf]

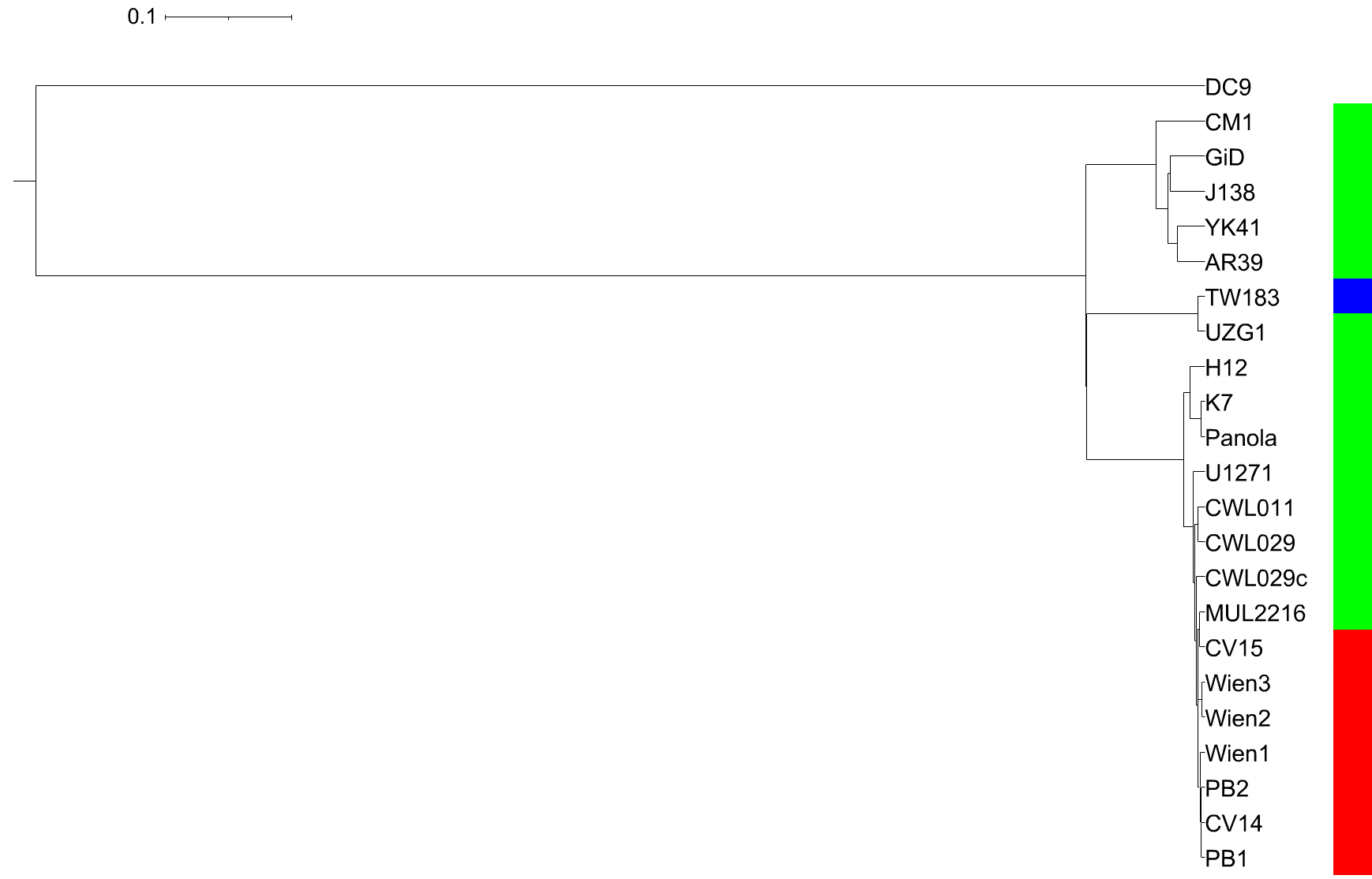

Supplement: Additional file 3: — ClonalFrame consensus phylogenetic tree. PNG image showing the consensus phylogenetic tree reconstructed by ClonalFrame. [file 12864_2015_1377_MOESM3_ESM.png]

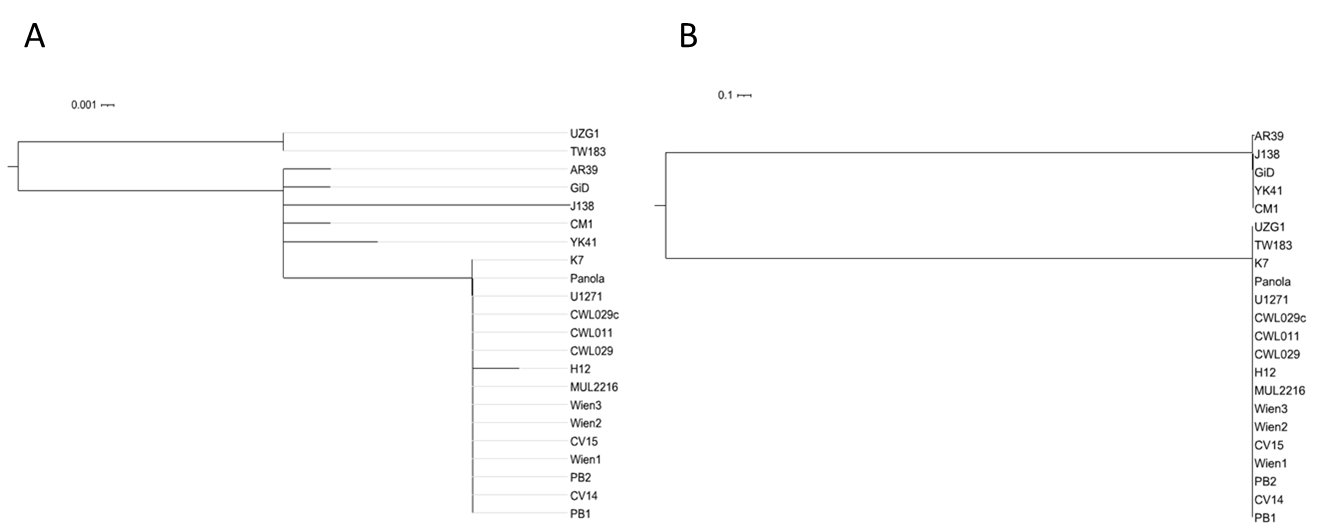

Supplement: Additional file 4: — Phylogenetic trees for recombined region CPn_0010.1. PNG image showing maximum likelihood phylogenetic trees for recombination-site 1; A: based on concatenated SNPs from genomic position 1 to 14 Kb and 16 Kb to 30 Kb; B: based on SNPs between genomic positions 14 Kb to 16 Kb. [file 12864_2015_1377_MOESM4_ESM.png]

A

0.1

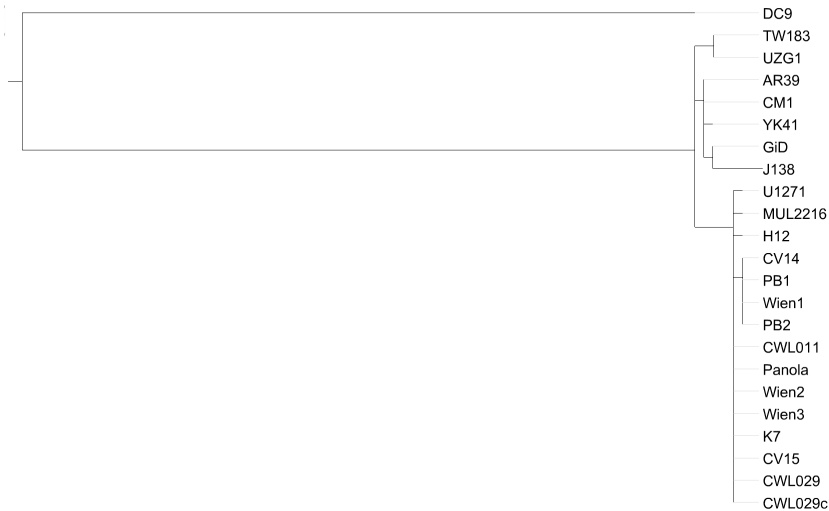

B

0.1

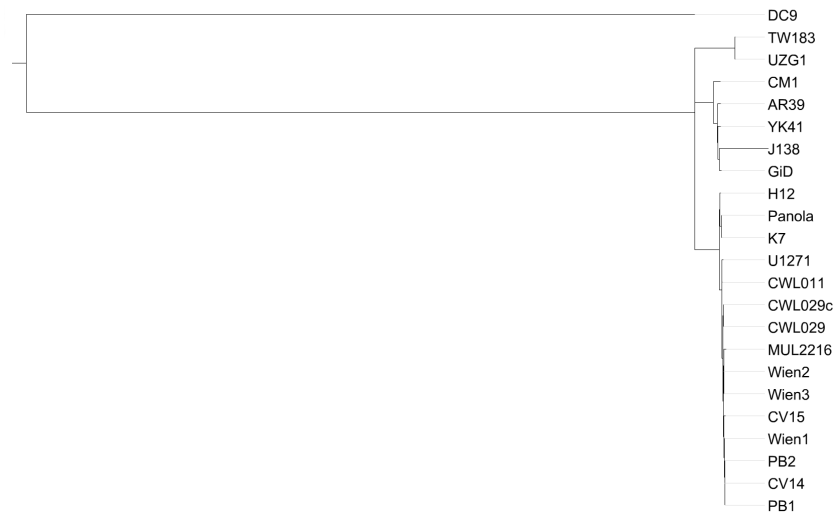

Supplement: Additional file 5: — Single nucleotide polymorphism phylogeny based on specific gene sets. PDF image showing maximum likelihood phylogenetic trees based on SNPs located in (A) 31 phylogenetic marker genes, (B) 545 genes that occur in all chlamydia. [file 12864_2015_1377_MOESM5_ESM.pdf]
